# Red Blood Cell Transfusion Independence Following the Initiation of Iron Chelation Therapy in Myelodysplastic Syndrome

**DOI:** 10.1155/2010/164045

**Published:** 2010-03-23

**Authors:** Maha A. Badawi, Linda M. Vickars, Jocelyn M. Chase, Heather A. Leitch

**Affiliations:** ^1^Department of Medicine, St. Paul's Hospital, The University of British Columbia, Vancouver, BC, Canada V6T1Z4; ^2^Department of Hematology, St. Paul's Hospital, The University of British Columbia, Vancouver, BC, Canada V6Z2A5

## Abstract

Iron chelation therapy is often used to treat iron overload in patients requiring transfusion of red blood cells (RBC). A 76-year-old man with MDS type refractory cytopenia with multilineage dysplasia, intermediate-1 IPSS risk, was referred when he became transfusion dependent. He declined infusional chelation but subsequently accepted oral therapy. Following the initiation of chelation, RBC transfusion requirement ceased and he remained transfusion independent over 40 months later. Over the same time course, ferritin levels decreased but did not normalize. There have been eighteen other MDS patients reported showing improvement in hemoglobin level with iron chelation; nine became transfusion independent, nine had decreased transfusion requirements, and some showed improved trilineage myelopoiesis. The clinical features of these patients are summarized and possible mechanisms for such an effect of iron chelation on cytopenias are discussed.

## 1. Introduction

The myelodysplastic syndromes (MDS) are characterized by ineffective hematopoiesis, cytopenias, and a risk of transformation to acute myeloid leukemia (AML); survival and AML risk are predicted by the International Prognostic Scoring System (IPSS) [1]. Because the median age of the MDS onset is in the seventh decade, most patients are ineligible for potentially curative hematopoietic stem cell transplantation [2]. Although other treatments are now available [3–7], the standard treatment for many MDS patients remains supportive care.

Most MDS patients eventually become red blood cell (RBC) transfusion dependent, risking iron overload [8], which may lead to cardiac, hepatic, and endocrine dysfunction. Recent studies suggest an adverse effect of RBC transfusion dependence on survival, predominantly in lower-risk MDS [9]. This effect was sufficiently significant that RBC transfusion dependence was incorporated into the World Health Organization Prognostic Scoring System (WPSS) for MDS [10]. 

While the benefits of iron chelation therapy are better established in thalassemia [11], recent retrospective studies in lower-risk MDS suggest a possible improvement in survival in transfusion dependent patients who received chelation [12]. Guidelines in MDS recommend chelation with an otherwise reasonable life expectancy and evidence of iron overload: elevated serum ferritin, iron related organ dysfunction, or chronic RBC transfusions [13, 14]. We present the clinical course of a RBC transfusion dependent MDS patient who became transfusion independent shortly after starting chelation and has remained transfusion independent for over three years. We review the literature on the abrogation of cytopenias in acquired anemias following chelation.

This paper was prepared in accordance with the requirements of the St. Paul's Hospital Institutional Research Ethics Board.

## 2. Case Report

A 76-year-old man was referred in June 2004. He was diagnosed with MDS in 1997 during a work-up of abnormal blood counts: white blood cells (WBC) 2.4 (normal 4.0–11.0) ×  10^9^/L, neutrophils 0.7 (2.0–8.0) ×  10^9^/L, hemoglobin (Hb) 133 (135–180) G/L, and platelets 108 (150–400) ×  10^9^/L. The following laboratory parameters were normal: creatinine, bilirubin, thyroid stimulating hormone, reticulocyte count, serum B12 level, red blood cell folate; and serum protein electropheresis. Bone marrow aspiration and biopsy showed refractory anemia (RA) by the French-American-British (FAB) classification [15] and cytogenetic analysis revealed trisomy 8 and loss of chromosome Y. Stem cell culture showed no erythropoietin independent colony growth, serum erythropoietin level was 148.3 (normal 3.3–16.6) IU/mL and IPSS score was intermediate-1. He remained transfusion independent until one month prior to referral, when the hemoglobin was 60 G/L, prompting the initiation of RBC transfusion support.

History and physical examination were otherwise unremarkable. WBC count at referral was 3.4 × 10^9^/L, Hb (transfused) 86 G/L, mean cellular volume (MCV) 121 fl (80–100), and platelets 44 × 10^9^/L. Serum ferritin was 1293 (15–370) ug/L with no prior ferritin levels available. Bone marrow aspiration and biopsy confirmed RA/refractory cytopenia with multilineage dysplasia (RCMD) by World Health Organization (WHO) criteria [16]. Marrow blast count was 2%.

Over a 30-month period, he required transfusion of 3 RBC units every 4 weeks to maintain the hemoglobin above 90 G/L and he complained of fatigue and functional limitation; he received approximately 90 RBC units in total. In January 2005, the ferritin was 2197 ug/L but he declined deferoxamine; however, in September 2006, he agreed to start deferasirox. Bone marrow aspiration and biopsy showed unchanged RCMD and karyotype. Deferasirox was started at 20 mg/kg/day. He required several dose interruptions and adjustments for renal insufficiency (peak creatinine 141 umol/L, normal to 100 umol/L) and the dose of deferasirox was titrated between 5–30 mg/kg/day. He received no other treatment for anemia.

**Figure 1:**
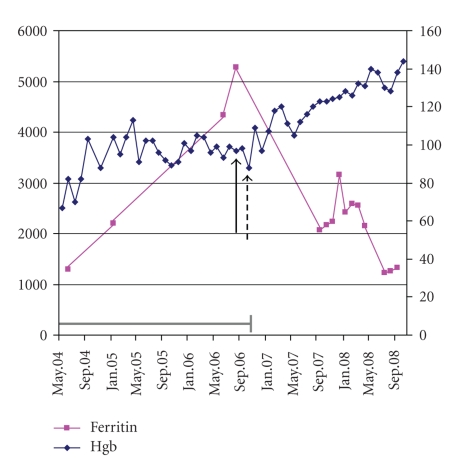
Hemoglobin and serum ferritin levels for a patient with MDS receiving iron chelation therapy. The solid black arrow represents the date at which chelation was initiated and the dashed arrow represents the date of his last red blood cell transfusion. The grey bar indicates the period during which transfusion requirement was 3 red blood cell units every 4 weeks.

Two months after starting chelation, the hemoglobin increased to 109 G/L and he has not required transfusion since. Mean hemoglobin over 24 months was 122 (range 96–144) G/L. Hemoglobin and ferritin levels are shown in Figure 1.The patient reports excellent energy and a significantly improved quality of life.

In May 2008, he was assessed for skin nodules and reported having similar nodules that appeared and regressed spontaneously for at least two years. A biopsy revealed leukemia cutis (LC). Despite this, he remained clinically well and transfusion independent for 17 months since the diagnosis of LC, over 41 months since the initial appearance of nodules, and 40 months since the initiation of chelation.

Characteristics of ten MDS patients, including ours, achieving transfusion independence with chelation are summarized in Table 1 [17–19]. Nine other patients with significant improvement in hemoglobin with chelation have been reported [19, 20]. Several features of these latter patients were not reported; however, eight received deferoxamine and one deferasirox, and the median time to improvement in RBC transfusion requirement was 14.4 (3–24) months. None of these patients were reported to have received any MDS treatment other than chelation.

## 3. Discussion

It is well established that chelation extends the survival of transfusion dependent patients with thalassemia by mitigating iron toxicity [21–24]. Recent retrospective data suggest a possible association between chelation and improved survival in MDS [12, 25]. The first report of decreased transfusion requirements with chelation was in 1990 [26]. Since then, nineteen MDS patients, including ours, are reported who had an improvement in hemoglobin or decreased transfusion requierements.

Our patient was transfusion independent within two months of starting chelation. The ferritin level decreased from 5271 to 1225 ug/L but remains elevated. Once transferrin is saturated, non-transferrin bound iron (NTBI) may be detected [27], correlating with the presence of potentially cytotoxic reactive oxygen species (ROS) [28]. Whether oxidative stress was present in our patient is unknown as few transferrin saturations were recorded and NTBI and ROS measurement are not readily available. However, the elevated ferritin over a long course despite chelation while transfusion independent may indicate a significant iron load, potentially leading to marrow toxicity and suppression of hematopoiesis. 

A patient with primary myelofibrosis (PMF) was reported whose hemoglobin increased from 76 ± 10 G/L to 100 G/L after starting chelation [19]; it returned to baseline (80 G/L) when chelation was interrupted, and increased again to 100 G/L when chelation resumed. A second PMF patient with baseline hemoglobin 60 G/L requiring 2 RBC units every two weeks achieved long-term transfusion independence one month after beginning deferiprone [29]. A third PMF patient with baseline hemoglobin 50–60 G/L requiring 2-3 RBC units per month became transfusion independent two months after starting deferasirox, an effect which persisted two years after chelation was stopped for improvement in ferritin (953 ug/L) and transferrin saturation (45%) [30]. These patients received no other treatment for PMF. A patient with aplastic anemia (initial Hb 45 G/L, neutrophil count 0.3 × 10^9^/L, and platelet count 3 × 10^9^/L) had trilineage recovery and became RBC transfusion independent after four years of deferoxamine [31]; this patient received low-dose erythropoietin following an initial improvement in blood counts. 

An improvement in MCV, platelet and white blood cell counts was also noted [18, 20]. In a report of six patients, two with pancytopenia had significant increases in WBC, neutrophil, and platelet counts (*P* ≤ .001) [20], seen within 3 months, maximized by 18 months, and in some patients, the effect persisted after chelation was discontinued. All of them had an elevated MCV prior to chelation, which decreased in five and normalized in two, suggesting possible improvement in erythropoiesis outside the MDS clone. In a report of eleven patients, the neutrophil count increased in eight of nine, and the platelet count in seven of eleven [18]. In our patient, recent WBC counts range between 3.1–4.3 × 10^9^/L and platelets consistently clump; the MCV is unchanged at 120 fl. 

The mechanisms by which chelation may improve cytopenias are unclear; however, iron was recently shown to have a suppressive effect on erythroid progenitors in vitro [32]. Erythroid colony assays on 42 MDS patients showed, in patients with ferritin 250 ug/L or more, that BFU-E were a mean of 2.35 (range 0–27) colonies per culture, compared to 10.1 (0–76) in patients with normal ferritin (*P* < .004); whether this is an effect of iron or due to other factors awaits further study. 

Although chelation may exert its protective effect by reversing the deposition of iron [23, 33], oxidative stress from iron overload may damage lipids, proteins, and nucleic acids [27, 28, 34–37], and it would be interesting to determine whether the protective effect of chelation on BFU-E might be from oxidative stress alleviation. A study of 15 patients with lower-risk MDS showed a decrease in RBC ROS following three months of chelation [38] and a relationship between ferritin and ROS content of CD34+ cells in MDS patients was established [39]. In thalassemia, chelation reduced oxidation in RBC and increased half-life from 12.1 ± 2.4 to 16.4 ± 4.3 days [40]. In the US03 trial of deferasirox in MDS, hematologic improvement was seen in 5 of 53 patients (9.4%) [41] and LPI, an indicator of oxidative stress, normalized over 12 months of chelation; whether this accounts for the mitigation of cytopenias remains to be determined. Finally, there are reports of increased erythropoietin levels with chelation in normal volunteers and this could contribute to an improvement in hemoglobin in MDS [3, 42].

It has been suggested that the transcription factor NF-*κ*B may be important in modifying myelopoiesis with chelation. In mononuclear cells of MDS patients [43], deferasirox induced a significant reduction in NF-*κ*B activity, but the opposite effect was seen with deferoxamine and deferiprone and no difference was noted in patients with or without iron overload. Although these findings might explain an effect of deferasirox on cytopenias, the effect of deferoxamine and deferiprone is not accounted for [18]. 

Sloand et al. showed improvement in erythropoiesis within the MDS clone in patients with trisomy 8 responding to immunosuppressive therapy [44]. While our patient has +8, no therapy other than chelation was administered; however, the MCV remains elevated, possibly indicating a significant contribution to erythropoiesis by the MDS clone. In the Jensen study, two of eleven patients had +8; in the first, the +8 clone decreased from 60% to 10% with chelation, and the second had persistence of +8 and clonal evolution to a deletion of 5q as well. Thus, immunomodulation resulting in improvement of erythropoiesis cannot be invoked as a predominant mechanism for transfusion independence in these patients.

## 4. Conclusions

In summary, a number of patients with acquired anemias have been reported in whom an improvement in cytopenias was seen following the initiation of iron chelation therapy, clinically manifested as a decrease in RBC transfusion requirements or even transfusion independence. This may occur in up to 9% of MDS [41] and possible mechanisms include reducing oxidative stress, altering intracellular levels of NF-*κ*B; increasing erythropoietin levels, or other mechanisms yet to be elucidated. In future trials of chelation, consideration could be given to including measures of these parameters, and conversely, trials of medications known to induce transfusion independence in MDS such as immunomodulatory, demethylating, or erythropoiesis stimulating agents could compare these in responders and nonresponders. Patients with iron overload considered for chelation should be assessed and monitored by a physician experienced with chelation medications.

##  Conflict of Interest

 HL and LV have received honoraria and research funding from Novartis Canada. All data collection and manuscript preparation were performed independent of financial support. MB and JC have no conflict of interest to disclose.

## Figures and Tables

**Table 1:** Clinical characteristics of 10 MDS patients achieving red blood cell transfusion independence with iron chelation therapy.

Clinical Feature (units)	*n*
Age at MDS diagnosis (years)	Median 58 (range 18–74)
Gender M : F	5 : 5
MDS subtype (FAB or WHO)	
RA	5
RARS	2
RCMD	1
RAEB	2
IPSS score	
Low	1
Intermediate-1	5
Intermediate-1 or 2	1
High	1
Not available	2
Iron chelation agent	
Deferoxamine	7
Deferasirox	3
Time to RBC transfusion independence (months)	Median 17.5 (range 1–24)
Duration of RBC transfusion independence (months)	Median 13 (range 3–40)

Abbreviations: F: female; FAB: French-American-British; IPSS: International Prognostic Scoring

System; M: male; *n*: number; RA: refractory anemia; RARS: refractory anemia with ringed sideroblasts,

RAEB: refractory anemia with excess blasts; RBC: red blood cell; RCMD: refractory cytopenia with multilineage dysplasia; and WHO: World Health Organization.
